# 83 The Impact of Tracheostomy on Long-term Patient Outcomes: A Burn Model System National Database Study

**DOI:** 10.1093/jbcr/irac012.086

**Published:** 2022-03-23

**Authors:** Kevin E Galicia, John Kubasiak, Anupama Mehta, Karen J Kowalske, Nicole S Gibran, Barclay T Stewart, Steven E Wolf, Colleen M Ryan, Jeffrey C Schneider

**Affiliations:** Loyola University Medical Center, Maywood, Illinois; Loyola University Medical Center, Maywood, Illinois; Brigham and Women's Hospital, Quincy, Massachusetts; Department of Surgery, The University of Texas Southwestern Medical Center, Dallas- Fort worth, Texas; University of Washington, Wellfleet, Massachusetts; University of Washington, Seattle, Washington; University of Texas Medical Branch at Galveston, Galveston, Texas; Harvard Medical School, Boston, Massachusetts; , Massachusetts

## Abstract

**Introduction:**

Management of the upper airway is crucial to burn care, especially in the setting of inhalation injury or burns to the face or neck. Endotracheal intubation is often performed to secure the airway; however, tracheostomy may be necessary in patients requiring prolonged ventilatory support. This study compares long-term outcomes of burn patients with and without tracheostomy.

**Methods:**

Data from the Burn Model System National Database, collected from 2013 to 2020, were analyzed. Demographic and clinical data were compared between those with and without tracheostomy. The following patient-reported outcome measures, collected at 6-, 12-, and 24-months, were analyzed: Veterans Rand 12 Physical Component Summary Score (VR-12 PCS), Veterans Rand 12 Mental Component Summary Score (VR-12 MCS), Satisfaction with Life (SWL), Community Integration Questionnaire (CIQ), Patient-Reported Outcomes Measurement Information System (PROMIS-29), employment status, and number of days to return to work. Regression models were used to assess the impact of tracheostomy status on long-term outcome measures, controlling for demographic and clinical variables.

**Results:**

Of the 714 patients included in this study, 39 (5.46%) received a tracheostomy and 675 (94.54%) did not. The two groups were similar across all demographic data collected. Tracheostomy patients were more likely to have flame injury, inhalation injury, larger burn size, more trips to the operating room, longer hospital stay, and greater number of days on a ventilator (p< 0.001). Regression model analyses demonstrated that tracheostomy was associated with worse VR-12 PCS scores at 6-, 12-, and 24-months (6.6 [95% CI 1.5, 11.8], p=0.012; 11.5 [6.2, 16.8], p< 0.001; 10.8 [4.2, 17.5], p=0.001). Tracheostomy was also associated with worse scores in two PROMIS-29 domains, physical function and pain interference. For physical function, the association was seen at 6-, 12-, and 24-months (7.4 [3.0, 11.8], p=0.001; 9.6 [5.2, 14.0], p< 0.001; 11.3 [5.8, 16.9], p< 0.001). For pain interference, the association was only seen at 12-months (-5.3 [-10.0, -0.55], p=0.029).

**Conclusions:**

After burn injury, patient-reported outcome measures of physical function and pain interference were significantly worse with tracheostomy.

